# COVID-19 and male infertility: An overview of the disease

**DOI:** 10.1097/MD.0000000000029401

**Published:** 2022-07-08

**Authors:** Mohammed Imad Malki

**Affiliations:** a College of Medicine, QU Health, Qatar University, Doha, Qatar.

**Keywords:** ACE-2, COVID-19, infertility, SARS-CoV-2

## Abstract

Since SARS-CoV-2 infection was first discovered in December 2019 in Wuhan City in China, it spread rapidly and a global pandemic of COVID-19 has occurred. According to several recent studies on SARS-CoV-2, the virus primarily infects the respiratory system but may cause damage to other systems. ACE-2, the main receptor for entry into the target cells by SARS-CoV-2, was reported to abundantly express in testes, including spermatogonia, Leydig and Sertoli cells. Nevertheless, there is no clinical evidence in the literature about whether SARS-CoV-2 infection has an impact on male reproductive health. Therefore, this review highlights the effect of SARA-CoV-2 infection on male reproductive health, including the reproductive system and its functioning, as well as gamete and male gonadal function that might be affected by the virus itself or secondary to immunological and inflammatory response, as well as drug treatments and the psychological stress related to panic during the COVID-19 outbreak.

## 1. Introduction

In late 2019, numerous cases of pneumonia caused by a novel corona virus (SARS-CoV-2) were reported in Wuhan city in China.^[1]^ Subsequently, the infection has spread worldwide causing acute respiratory distress syndrome (SARS) termed as “COVID-19” by the World Health Organization (WHO). The quick spread of this novel virus all over the world caused in the declaration of a pandemic by the WHO leading to massive changes in social behaviors.^[1]^ COVID-19 has had a disturbing impact on many of those suffering with the condition. It also had a significant impact on healthcare delivery for all patients without COVID-19.

Signs of SARS-CoV-2 infection including cough, shortness of breath, fever, fatigue, headache, sputum production, and myalgia. Additionally, patients reported gastrointestinal symptoms or anosmia.^[2]^ The rigorousness of infection varieties from presymptomatic cases, to asymptomatic cases, to a flu-like disease, to serious illness and death. Patients in critical condition may suffer from respiratory failure, shock, or multiorgan dysfunctions. About 75% to 80% of COVID-19 infections are mild with only flu-like symptoms, 15% to 20% of infections are severe, requiring hospitalization for supplemental oxygen, and 5% of those are critical, requiring mechanical ventilation.^[3]^ The risk factors for the fatal illness including age and underlying medical conditions such as diabetes, chronic respiratory disease, cardiovascular disease, hypertension, and cancer.^[4]^ Fatality accounted up to 3% of infections and commonly occur in individuals over the age 60 or those with underlying medical conditions. However, it also can affect younger persons, possibly related to the inoculum.

The role of SARS-CoV-2 infection in human male reproductive system has yet to be fully understood. Therefore, the aim of this review is to highlight what is currently known about the effect of the novel SARS-CoV-2 infection on male reproductive system.

## 2. Pathophysiology of COVID-19

**Figure 1.**
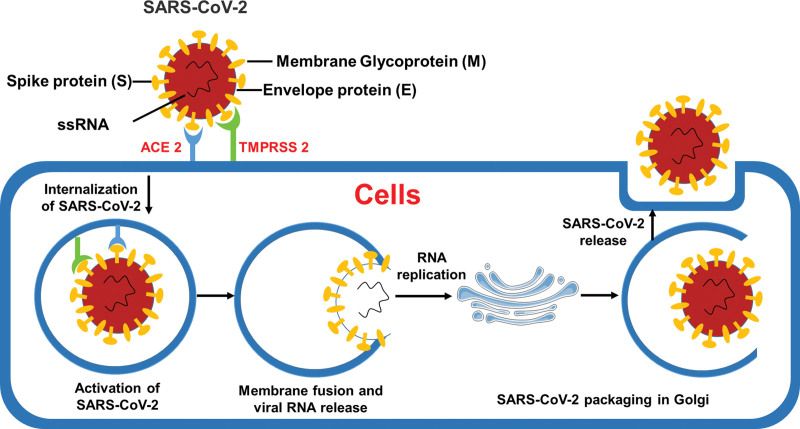
Possible pathophysiology of SARS-CoV-2. Binding of SARS-CoV-2 to its receptors ACE-2 and TMPRSS 2. Upon binding to these receptors, the virus fuse with the membrane and enter the cells, followed by translation and replication of the proteins. The replication complex, which makes more RNA of the SARS-CoV-2 are packaged in Golgi. SARS-CoV-2 release outside to spread to other cells.

The novel corona viruses belong to Coronaviridae family that are enveloped viruses with a positive-sense single-strand RNA of around 32 kb. The viral molecules comprise 4 main structural proteins including the spike, membrane, envelope protein, and nucleocapsid. The spike protein protrudes from the envelope of the virion that described to play a critical role in the receptor host selectivity and cellular adhesion. Recent studies reported that SARS-CoV and SARS-CoV-2 spike proteins interacted with angiotensin-converting enzyme 2 (ACE-2)^[5,6]^ (Fig. 1). In addition, other cellular receptors also reported to play a secondary role in the viral adhesion such as the C-type lectin CD209L and DC-SIGN binds to SARS-CoV. Nevertheless, ACE-2 appears to be the vital functional receptor for the SARS-CoV^[7,8]^ and probably for SARS-CoV-2.^[9]^ The interaction between the viral protein and its receptor in the cell membrane is a key process in the replication cycle. Moreover, the efficiency of viral infection is significantly dependent on this process.

Transmission of SARS-CoV-2 to extrapulmonary organs remains unclear. SARS-CoV-2 infectious virus molecules were isolated from respiratory samples,^[1]^ in addition to fecal^[10]^ and urine^[11]^ specimens from COVID-19 patients, suggesting that numerous organ dysfunction in fatal COVID-19 patients that might cause by a direct attack from the virus. Moreover, a recent study based on a clinical sample reported that about 1% of blood samples had positive PCR test results, suggesting that SARS-CoV-2 infection might be a systemic.^[10]^ This may indicate that SARS-CoV-2 can spread via blood route to other organs in the body except the lungs. However, the exact mechanism needs to be further examined.^[11]^

## 3. Effect of COVID-19 on male reproductive system

**Figure 2.**
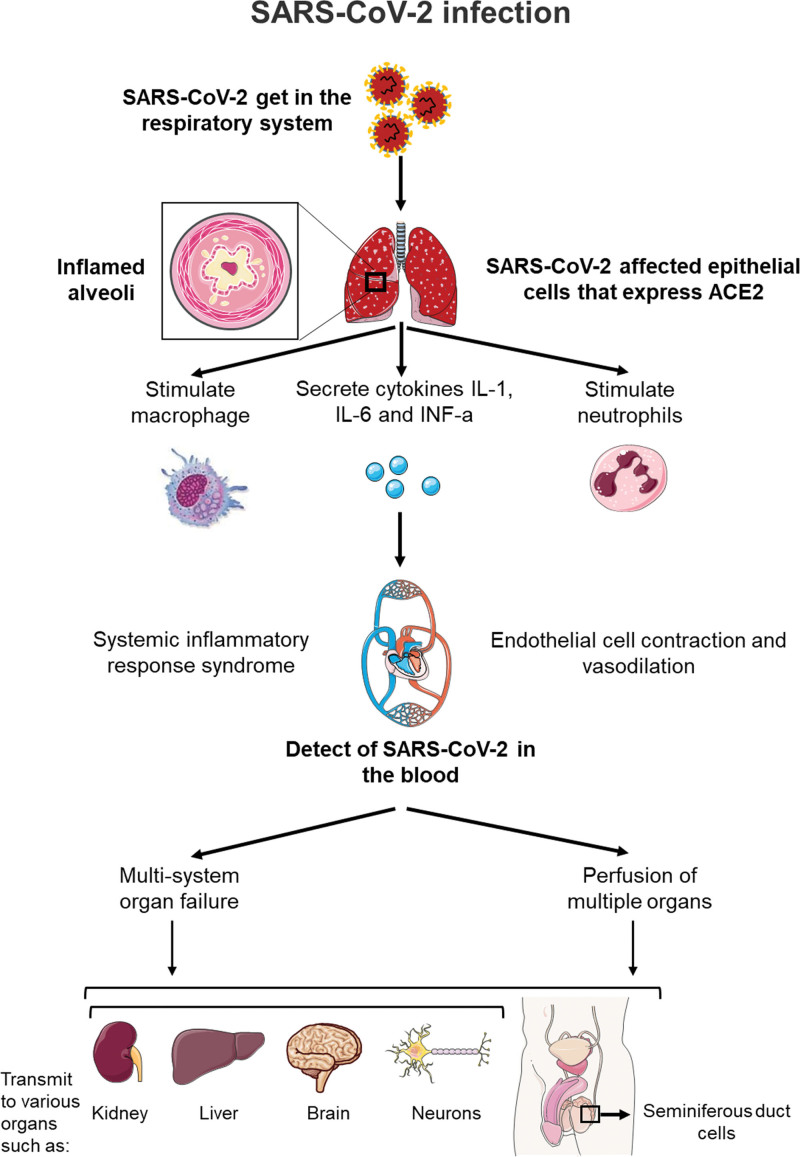
After SARS-CoV-2 enters into the respiratory system, it causes an inflammation of alveoli that at certain stage stimulates macrophages, neutrophils and secrete cytokines. Severe lung inflammation leads to systemic inflammatory response syndrome and endothelial cell contraction and vasodilation. SARS-CoV-2 becomes detectable in the blood and capable of casing, multi-system organ failure and perfusion of multiple organs express AEC2 such as Kidney, Liver, Brain, Neurons and seminiferous duct cells in the testis.

The effect of SARS-CoV-2 infection upon human male reproductive systems still to be fully explained. However, reports from other coronavirus family, specifically SARS-CoV, contributed in explaining the possible pathway of tissue-specific viral pathophysiology. As mentioned previously, ACE-2 receptors reported to play a significant role in the pathogenesis of COVID-19 infection. Upon binding of SARS-CoV-2 virus to ACE-2 receptors will facilitate its entry and replication in the cell.^[2]^ Thus, cells that showing high level of ACE-2 expression might be targeted and damaged by the virus^[12]^ (Table 1; Figure 2). Several studies have detected a high ACE-2 expression level in testicular cells, predominantly in spermatogonia, seminiferous duct cells, Sertoli cells and Leydig cell.^[30,31]^ According to the results of these studies, it reported that both testis might be a potential target for direct damage by SARS-CoV-2 virus. Therefore, the spermatogenesis process could be affected, posing the risk to male fertility. A recent study showed that ACE-2 was mainly expressed in spermatogonia, Leydig and Sertoli cells in the human testes. ACE-2-positive spermatogonia significantly expressed a higher number of genes related to viral reproduction and transmission, and a lower number of genes associated with spermatogenesis compared with ACE-2-negative spermatogonia. ACE-2-positive Leydig and Sertoli cells significantly expressed higher genes that engaged in cell-cell junction and immunity, and lower genes related to mitochondria and reproduction.^[32]^ These results may clearly suggest that the testes are a high-risk organ vulnerable to SARS-CoV-2 infection leading to spermatogenic failure. Remarkably, the expression of ACE-2 in testes is age related.^[31]^ The highest expression of ACE-2 was reported in 30-year-old patients, which is higher than those in their twenties, while 60-year-old patients showed the lowest level of expression of ACE-2.^[14]^ This may suggest that young male patients are at higher risk of testicular dysfunction by COVID-19 infection than older male patients. Another study carried out in 2002 subsequent the outbreak of SARS-CoV infection, reported that orchitis was a recognized complication of SARS-CoV,^[33]^ as demonstrated by massive IgG precipitation in testicular interstitial tissue leading to destruction of germ-cell and extensive testicular leukocyte infiltration. Remarkably, in situ hybridization did not detect any viral genomic materials in specimens of the testicular tissue.^[33]^ This suggests that testicular dysfunction is due to inflammatory and immunological response instead of a direct damage from the virus. The main question to be answered that whether COVID-19 playing a role in causing testicular damage and male infertility. To date, there is no exact answer as studies of follow-up of reproductive function of recovered male patients is still required.

**Table 1:** Expression of ACE-2 in various human tissues

Tissues	Organ/systems	References
Alveolar epithelial cells	Lung	^[13,14]^
Enterocytes	Small intestine	^[15–17]^
Arterial smooth muscles	Blood vessels	^[13]^
Cholangiocytes	Bile duct/Liver	^[18,19]^
Islets	Pancreas	^[20,21]^
Myocytes and Fibroblasts	Cardiovascular	^[14,22,23]^
Proximal Tubule	Renal	^[14]^
Urothelial	Bladder	^[14,24]^
Mucosa	Oral cavity	^[25]^
Ovary, uterus, vaginal and placental tissues	Female reproductive system	^[26–29]^

### 3.1. Effect of COVID-19 on male gametes

Currently, there have been no definite reports of the virus in the male gametes that might be impacted directly by infection with SARS-CoV-2 or other coronavirus subtypes. Nevertheless, there is a clear evidence that fever can affect the spermatogenesis. Thus, male fertility can be diminished for 72 to 90 days subsequent to COVID-19 infection that caused by a significant decrease in sperm concentration and motility.^[34,35]^ The current regulations stated that gametes have been isolated from patients with other viral diseases including hepatitis and HIV should be treated with special precautions to reduce exposure of the noninfected partner and cross-contamination of reproductive tissues in the laboratory.^[36]^ To date, there is no clear regulations for screening sperm donors for SARS-CoV-2. Therefore, further investigation is required to guarantee the safety of stored gametes and the safety of patients that requiring assisted reproduction.

## 4. Effect of COVID-19 treatment on male reproductive system

Up to date, drugs for COVID-19 treatment including mainly antiviral drugs (such as ribavirin, interferon, and lopinavir/ritonavir), antibiotics (such as azithromycin and moxifloxacin) and steroidal drugs such as glucocorticoids.

Ribavirin is a broad-spectrum antiviral drug. A study based on animal experiments showed that ingestion of ribavirin significantly decreased the concentrations of testosterone and impaired spermatogenesis.^[37]^ It also caused sperm abnormalities in rats.^[38]^ However, this toxic effect was revocable.^[39]^ Clinical studies showed that ribavirin combined with interferon treatment leading to male infertility by decreasing the sperm count.^[40,41]^ Pharmacokinetic studies illustrated that drug concentration in seminal plasma within serum antiviral therapy was a twice higher than that in the serum. Thus, contraception was strongly advisable through the period of medication.^[42]^ Furthermore, ribavirin caused sperm DNA fragmentation for up to 6 months.^[43]^ Hence, contraception was also advisable for a period after antiviral treatment cessation.

Lopinavir/ritonavir reported to impair spermatogenesis in rats, probably by oxidative damage.^[44]^ Chloroquine phosphate has also impaired spermatogenesis and epididymal function in male rats.^[45]^ Further studies require to explore the effect of Arbidol upon reproductive system.

Glucocorticoids can cause expansion in interstitial space of the spermatogenic epithelium, damage cell connections, and disturb the blood-testis barrier causing harmful substances to enter testicular tissue.^[46,47]^ Glucocorticoids reported to cause germ-cell apoptosis via receptors on germ cells.^[47]^ Therefore, glucocorticoids are only recommended for a short period in COVID-19 patients treatment who suffer from a rapid progress on imaging manifestations, progressive deterioration of oxygenation indicators, and massive activation of inflammatory reactions in the body. Minimum doses within a short period have little effect on male reproductive system.

## 5. Effect of COVID-19 on outbreak panic

Several studies showed that the epidemic of prominent infectious diseases (such as SARS, H1N1 virus, and Middle East respiratory syndrome [MERS]) correlated with psychological stress associated with excessive anxiety among patients, frontline health workers as well as public such as fear, depression, and post-traumatic stress disorder (PTSD).^[48–51]^ This might lead to gradual distraction of the homeostasis of the body and constant activation of the central stress response system (mainly regulated through the hypothalamic-pituitary-adrenal [HPA] axis). Despite of the acute HPA response to stressors by a mechanism of self-protective, continuous activation of the HPA axis by excessive panic might cause downregulation of the HPA axis that can obstruct the reproductive function of the body as well as alter fetal development that might lead to poor reproductive outcomes.^[52]^ Moreover, there are sex differences in the regulation of stress response, primarily due to the interaction between the HPA axis and the hypothalamic-pituitary-gonadal axis. This interaction might lead to irregularities of stress responses, and the latter could sequentially affect the former and thus aggravate psychological disorders.^[53]^ Hence, there are many complicated mechanisms in the interactions between psychological disorders and stressors. Thus, it is necessary to illustrate the impacts of panic psychology upon male reproductive health during the current outbreak of COVID-19.

There is an increasing evidence on the relation between male infertility and psychological disorders. Interestingly, it reported that female partners of men with psychological disorders such as major depression were unlikely to achieve conception compared with those of men without major psychological disorders.^[54]^ Poor fertility performance throughout a period of psychological disorders was associated with oligospermia leading to sexual dysfunction. Stress and negative moods such as depression and anxiety are capable to impact the seminal parameters both at macroscopic and cellular levels such as oligospermia, azoospermia, hypospermia. In addition to increase in sperm DNA fragmentation.^[55–57]^

In addition to poor sperm quality, the other significant cause of the poor fertility performance among men with psychological stress is sexual dysfunction such as low libido, homosexuality and erectile difficulties. For example, a model of sexual dysfunction in PTSD was established by Yehuda et al It was developed by an inability to regulate and redirect the physiological arousal that are needed for healthy sexual function.^[58]^ Similarly, it reported that sexual dysfunction was also associated with other negative moods including depression, anxiety, and fear.^[59]^

## 6. Conclusion

Human spermatogenesis is organize and dynamic process of cell differentiation that maintained by self-renewal and differentiation of spermatogonial stem cells (SSCs). It is strictly controlled by a dynamic microenvironment in seminiferous tubules of the testicular. Sertoli cells of the testicles are the only somatic cell type in the tubules that precisely collaborate with spermatogenic cells to control spermatogenic cell differentiation via paracrine signaling.^[60]^ The Leydig interstitial cells are found adjacent to the seminiferous tubules in the testicle. They produce testosterone in the support of luteinizing hormones to maintain the differentiation of spermatogenic cells.^[61]^ Any functional deformities in male germ cells or in those supporting somatic cells will lead to spermatogenic failure and poor male fertility.

The outbreak of COVID-19 is quickly spreading worldwide. As of June 2020, there were over 8 million confirmed cases of COVID-19 worldwide, and the total number of deaths from this disease had exceeded 400,000. There are a rising concern by several studies on impact of COVID-19 upon male reproductive health. Based on the little evidence to date, it can be proposed that the possible pathogenicity and attack of COVID-19 on testicular tissues might affect testicular spermatozoa quality leading to poor male fertility. Special attention should be focused to evaluate an appropriate intervention in the young couples fertility throughout and after this outbreak, specifically for those infected with SARS-CoV-2 virus.

As is common of this family of viruses, binding of SARS-CoV-2 to its receptors ACE-2 and TMPRSS 2 that the virus fuse with the membrane and enter the cells, followed by translation and replication of the proteins. The replication complex, which makes more RNA of the SARS-CoV-2 are packaged in Golgi. SARS-CoV-2 release outside to spread to other cells. It is a matter of concern, evidence exists that correlated coronaviruses with severe orchitis. Whereas sperm counts can be diminished by high temperature alone, the question of other possible long-standing effects on male gametes is critical. Specially, whether the shedding of virus in some individuals, which may impact the safety and storage of gametes.

In conclusion, there is an excessive possibility of testicular damage and later infertility following COVID-19 infection. The probability of testicular damage might be caused by either direct viral invasion via binding of SARS-CoV-2 virus to ACE-2 receptors or secondary to immunological and inflammatory response. Therefore, follow-up studies of reproductive function of recovered male patients are necessary to investigate this possibility.

## Acknowledgments

The author is grateful for S. Rostom for stimulating discussions and J. Alhmoud for illustration design.

## Author contribution

The author confirms being the sole contributor of this work and has approved it for publication.
